# Adhesion of *Staphylococcus aureus* to *Candida albicans* During Co-Infection Promotes Bacterial Dissemination Through the Host Immune Response

**DOI:** 10.3389/fcimb.2020.624839

**Published:** 2021-02-02

**Authors:** Katrien Van Dyck, Felipe Viela, Marion Mathelié-Guinlet, Liesbeth Demuyser, Esther Hauben, Mary Ann Jabra-Rizk, Greetje Vande Velde, Yves F. Dufrêne, Bastiaan P. Krom, Patrick Van Dijck

**Affiliations:** ^1^Laboratory of Molecular Cell Biology, Institute of Botany and Microbiology, Department of Biology, KU Leuven, Leuven, Belgium; ^2^VIB Center for Microbiology, Leuven, Belgium; ^3^Louvain Institute of Biomolecular Science and Technology (LIBST), UC Louvain, Louvain-la-Neuve, Belgium; ^4^Laboratory for Pathology, UZ Leuven and Department of Imaging and Pathology, Translational Cell and Tissue Research, KU Leuven, Leuven, Belgium; ^5^Department of Oncology and Diagnostic Sciences, Dental School, University of Maryland, Baltimore, Baltimore, MD, United States; ^6^Department of Microbiology and Immunology, School of Medicine, University of Maryland, Baltimore, Baltimore, MD, United States; ^7^Biomedical MRI/MoSAIC, Department of Imaging and Pathology, KU Leuven, Leuven, Belgium; ^8^Department of Preventive Dentistry, Academic Centre for Dentistry Amsterdam (ACTA), Vrije Universiteit Amsterdam and the University of Amsterdam, Amsterdam, Netherlands

**Keywords:** *Candida albicans*, *Staphylococcus aureus*, oral candidiasis, polymicrobial, adhesins, immune response, candidalysin

## Abstract

Interspecies interactions greatly influence the virulence, drug tolerance and ultimately the outcome of polymicrobial biofilm infections. A synergistic interaction is observed between the fungus *Candida albicans* and the bacterium *Staphylococcus aureus*. These species are both normal commensals of most healthy humans and co-exist in several niches of the host. However, under certain circumstances, they can cause hospital-acquired infections with high morbidity and mortality rates. Using a mouse model of oral co-infection, we previously showed that an oral infection with *C. albicans* predisposes to a secondary systemic infection with *S. aureus*. Here, we unraveled this intriguing mechanism of bacterial dissemination. Using static and dynamic adhesion assays in combination with single-cell force spectroscopy, we identified *C. albicans* Als1 and Als3 adhesins as the molecular players involved in the interaction with *S. aureus* and in subsequent bacterial dissemination. Remarkably, we identified the host immune response as a key element required for bacterial dissemination. We found that the level of immunosuppression of the host plays a critical yet paradoxical role in this process. In addition, secretion of candidalysin, the *C. albicans* peptide responsible for immune activation and cell damage, is required for *C. albicans* colonization and subsequent bacterial dissemination. The physical interaction with *C. albicans* enhances bacterial uptake by phagocytic immune cells, thereby enabling an opportunity to disseminate.

## Introduction

The fungus *C. albicans* is a commensal organism of the oral cavity, skin, gastro-intestinal and urogenital tracts (Berman, 2012; Mayer et al., 2013). However, the use of corticosteroids can trigger its transition to a pathogen. *C. albicans* virulence factors include morphogenesis, adhesion, invasion and biofilm formation (Mukaremera et al., 2017). One of the key players in *C. albicans* biofilm formation is the Als3 protein (Zhao et al., 2004; Nobile et al., 2006), a member of the agglutinin-like sequence (*ALS*) family which is only expressed during the hyphal or pseudo-hyphal phase (Hoyer et al., 2008; Hoyer and Cota, 2016). Another *ALS* family member, *ALS1*, shows 81% sequence homology to *ALS3* and has overlapping functions but different expression patterns (Hoyer et al., 1998; Liu and Filler, 2011; Ho et al., 2019). Remarkably, a *C. albicans ALS3* deletion strain is only impaired in biofilm formation *in vitro* but not *in vivo*, presumably due to the activation of additional adhesin genes, such as *ALS1* (Nobile et al., 2006).

Within the host environment, fungi predominantly co-exist with diverse micro-organisms in complex communities (Shirtliff et al., 2009; Peters et al., 2012a; Lohse et al., 2018; Sultan et al., 2018). In the oral cavity, *C. albicans* is known to interact with other commensal bacterial species and is often co-isolated with other pathogens (Montelongo-Jauregui and Lopez-Ribot, 2018). *S. aureus*, a Gram-positive commensal bacterium, mainly colonizes the nares and skin but is considered a member of the oral microbial flora as well (McCormack et al., 2015; Thomer et al., 2016). *S. aureus* has a wide variety of virulence factors, yet it is not invasive and thus requires a portal of entry to cause systemic infections (van der Mee-Marquet et al., 2004; David and Daum, 2010; DeLeo et al., 2010; Kong et al., 2016). It is well established that *C. albicans* and *S. aureus* display a synergistic interaction with important clinical and therapeutic implications such as higher mortality rates (Carlson, 1982; Carlson, 1983; Peters and Noverr, 2013; Kong and Jabra-Rizk, 2015; Carolus et al., 2019). For the *C. albicans ALS3* deletion strain, binding of *S. aureus* to the hyphae is reduced, indicating the importance of the Als3 protein in the interaction with *S. aureus* (Peters et al., 2012b).

Oropharyngeal candidiasis (OPC), primarily caused by *C. albicans*, is one of the most common fungal biofilm-like infections characterized by thick white patches on the tongue and inner cheeks (Fanning et al., 2012; Patil et al., 2015; Swidergall and Filler, 2017). The mouse model of OPC is an established model to study oral candidiasis due to the close similarity of the disease process and host immune response, to that in humans (Solis and Filler, 2012). The model was successfully adapted to study *C. albicans*-*S. aureus* interactions during oral co-infection (Kong et al., 2015; Schlecht et al., 2015; Vila et al., 2020). Using this model, we previously found that in contrast to animals only infected with *S. aureus*, those co-infected with *C. albicans* had a higher oral colonization of *S. aureus* and were susceptible to *S. aureus* systemic infection. Subsequent studies aiming to provide mechanistic insights into this phenomenon attributed a role for the *C. albicans* Als3 adhesin, since dissemination of *S. aureus* was reduced when co-infected with a *C. albicans ALS3* deletion strain (Kong et al., 2015; Schlecht et al., 2015). In addition, the immune response appears to be altered by co-infections of *C. albicans* and *S. aureus* compared to the corresponding single species infections and could be involved in facilitating fungal-bacterial synergy (Peters and Noverr, 2013; Allison et al., 2019). Interestingly, candidalysin, a peptide encoded by *C. albicans ECE1*, was recently shown to be the main factor involved in mucosal immune activation, neutrophil recruitment and cell damage during *C. albicans* infection (Moyes et al., 2016; Naglik et al., 2019; Rogiers et al., 2019). Candidalysin is one of eight peptides which are produced after processing of Ece1 by kexin. However, the role of this newly identified toxin in mediating interspecies interactions was not previously investigated.

In this study, we provide insights into the multifaceted and dynamic mechanisms leading to *S. aureus* dissemination during oral co-infection with *C. albicans*. We demonstrate that the interaction with *C. albicans* is essential for *S. aureus* to cause systemic disease and identified both Als3 and Als1 as important molecular players of this interaction. Furthermore, we found that the host immune response is a key factor required for bacterial dissemination. In this regard, we show that both the level of immunosuppression and secretion of the peptide toxin candidalysin play pivotal roles.

## Materials and Methods

### Strains and Growth Conditions

*C. albicans* and *S. aureus* strains used in this study are listed in **Table 1**. Prior to every experiment, *C. albicans* strains were cultured overnight in liquid yeast extract-peptone-dextrose (YPD) medium at 30°C and *S. aureus* in 0.6x tryptic soy broth (TSB) medium supplemented with 0.2% glucose at 37°C. For GFP-expressing *S. aureus*, the media was supplemented with 2 µg/ml of tetracycline. J774 murine macrophages (ATCC TIB-67) were maintained in Dulbecco’s modified Eagle’s medium (DMEM) with 10% fetal bovine serum (FBS) and antibiotic/antimycotic solution (PSA; 100 U/ml of penicillin, 100 µg/ml of streptomycin and 250 ng/ml of amphotericin B). For phagocytosis assays, 80% confluent macrophages were removed from culture flasks and 10-fold diluted in DMEM containing 10% FBS. HeLa cells (ATCC CCL-2) were maintained in DMEM containing 4.5 g/l glucose, supplemented with 1x GlutaMAX, 10% FBS, and 50 µg/ml of gentamycin.

**Table 1:** Strains used in this study.

Strain name	Genotype	Reference
***C. albicans* strains**		
SC5314	Wild type, clinical isolate	(Gillum et al., 1984)
*als1*Δ/Δ	SC5314 *als1-1*Δ*::FRT/als1-2*Δ*::FRT*	This study
*als3*Δ/Δ	SC5314 *als3-1*Δ*::FRT/als3-2*Δ*::FRT*	This study
*als1 als3*ΔΔ /ΔΔ	SC5314 *als1-1*Δ*::FRT/als1-2*Δ*::FRT* *als3-1*Δ*::FRT/als3-2*Δ*::FRT*	This study
*ece1*Δ/Δ	SC5314 ece*1-1*Δ*::FRT/ece1-2*Δ*::FRT*	This study
***S. aureus* strains**		
USA300	Wild type, clinical isolate	(Tenover and Goering, 2009)
USA300 - GFP	USA300 – pMV158 GFP	This study

For each of the deletion strains, three independent transformants were generated.

### Strain Construction

The SAT-flipping strategy was used for the construction of the *C. albicans* deletion strains in the SC5314 background (Sasse and Morschhauser, 2012). Briefly, deletion cassettes were constructed by insertion of an upstream and downstream fragment of the target sequence (**Supplementary Table 1**) in the pSFS2 plasmid containing a nourseothricin resistance marker and a site-specific recombinase under a maltose inducible promotor. Plasmids were transformed in *C. albicans* to obtain deletion of the first allele and correct transformants were selected on YPD plates containing 200 µg/ml of nourseothricin. Correct transformants were grown in YP medium containing 2% maltose for excision of the cassette. A second round of transformation and excision was performed to delete the second allele (Sasse and Morschhauser, 2012). Three independent transformants were generated for each deletion strain. Deletion of the gene was checked using PCR. To verify that these deletions did not affect the growth, growth curves were made in YPD at 30°C and RPMI medium (RPMI 1640 medium with l-glutamine (Sigma) and buffered with 0.165 M morpholinepropanesulfonic acid at pH 7) at 37°C by measuring the OD_600nm_ over time. For the construction of the GFP-expressing *S. aureus* strain, plasmid pMV158-GFP containing optimized GFP under control of the MalP promotor (Nieto and Espinosa, 2003) was introduced into *S. aureus* USA300 by electroporation and transformants were selected on TSB plates containing 10 µg/ml of tetracycline (Li et al., 2011).

### *In vitro* static *C. albicans* Adhesion and Biofilm Assay

Biofilm and adhesion assays were performed in flat bottom 96-well polystyrene plates. One day prior to the assay, plates were coated with 100 µl of FBS and incubated overnight at 37°C. Overnight *C. albicans* cultures were washed twice with phosphate buffered saline (PBS) and 1 × 10^6^ C*. albicans* cells in 100 µl RPMI were added to the wells. Plates were statically incubated for 90 min at 37°C to allow adhesion. For biofilm formation, wells were washed to remove non-adherent cells and supplemented with fresh RPMI medium after the 90 min adhesion period and further incubated for 24 h. Afterwards, the medium was removed, wells were gently washed twice with PBS and resuspended in 200 µl PBS. Wells were sonicated and plated on YPD plates in 10-fold dilutions for the enumeration of CFU.

### *In Vitro* Static Mixed Species Adhesion Assay

Mixed species adhesion assays were performed in flat bottom 96-well polystyrene plates. One day prior to the assay, plates were coated with 100 µl of FBS and incubated overnight at 37°C. Next, serum was removed and 1 × 10^6^ C*. albicans* cells in 100 µl RPMI were added to the wells. Plates were statically incubated for 90 min at 37°C to allow adhesion and germ tube formation of the *C. albicans* cells. Afterwards, the supernatant was removed and 1 × 10^6^ *S. aureus* cells in 100 µl RPMI were added to the wells. Plates were incubated at 37°C for 2 h to allow attachment of the bacterial cells to the *C. albicans* cells. Afterwards, the medium was removed, wells were gently washed twice with PBS and resuspended in 200 µl PBS. Wells were sonicated, 10-fold dilutions were prepared and plated on TSB plates containing 8 µg/ml of amphotericin B and YPD plates containing 10 µg/ml of vancomycin for the enumeration of CFU of *S. aureus* or *C. albicans*, respectively.

### *In Vitro* Dynamic Adhesion Assay Using a Bioflux System

A Bioflux 1000Z setup (Fluxion Biosciences, USA) was used with an Axio Observer Zeiss Z1 automated microscope. All solutions were pre-warmed at 37°C before use to prevent air bubbles. PBS supplemented with 10% FBS at 37°C was used to fill the channels of a 48-well microfluidics plate (Fluxion Biosciences, USA) from the inlet using a flow rate of 0.5 dyne/cm^2^ for 30 min. Afterwards, an inoculum of *C. albicans* 3 × 10^6^ cells/ml of in PBS was added to the channel from the outlet well at a flow rate of 0.5 dyne/cm^2^ until the complete channel was filled with cells. Cells were allowed to adhere to the surface for 30 min without flow. Yeast-nitrogen-base media (YNB; supplemented with 0.5% glucose at pH 7) was flowed from the inlet well at 0.5 dyne/cm^2^ for 3 h to allow hyphae formation. Next, a GFP-expressing *S. aureus* inoculum of 1 × 10^7^ cells/ml in PBS was added at a flow rate of 0.5 dyne/cm^2^ from the inlet well. Images were taken every minute for 90 min. *C. albicans* and *S. aureus* were visualized by imaging bright-field illumination and the GFP signal at 20x magnification on three randomly selected channel positions per condition. Images were analyzed using the ImageJ software (Schlecht et al., 2015).

### Single-Cell Force Spectroscopy

*S. aureus* cells were grown overnight in TSB at 37°C, 150 rpm. The cells were harvested in the stationary phase and washed three times with PBS. *C. albicans* cells were incubated in RPMI medium to induce hyphae formation. Germinating *C. albicans* cells were immobilized on hydrophobic substrates. To prepare those substrates, glass coverslips coated with a thin layer of gold were immersed overnight in a 1 mM solution of 1-dodecanethiol, rinsed with ethanol and dried under N_2_. After induction of germ tube formation, 200 µl drops of the concentrated suspension were deposited on the hydrophobic substrates and let stand for 3 h. The substrate was then rinsed to remove loosely attached cells and transferred to the AFM setup. For single-cell experiments, colloidal probes were prepared by attaching single silica microsphere (6.1 μm diameter, Bangs laboratories) with a thin layer of UV-curable glue (NOA 63, Norland Edmund Optics) on triangular shaped tip-less cantilevers (NP-O10, Bruker) using a Nanowizard IV AFM (JPK Instrument, Berlin, Germany). Cantilevers were then immersed for 1 h in Tris-buffered saline (Tris, 50 mM; NaCl, 150 mM; pH 8.5) containing 4 mg/ml of dopamine hydrochloride, rinsed in Tris-buffered saline, and used directly for cell probe preparation. In this end, 50 μl of a 100-fold diluted solution of *S. aureus* were transferred in a petri dish and the bacteria were let to settle for 15 min. The nominal spring constant of the colloidal probe was determined by the thermal noise method. The colloidal probe was then mounted into the AFM and brought into contact with an isolated bacterium. The bacterial probe was positioned over an immobilized *C. albicans* germ tube on the hydrophobic surface. AFM measurements were performed at room temperature in PBS buffer using a Nanowizard 3 and Nanowizard 4 AFM (JPK Instrument, Berlin, Germany). Using the inverted optical microscope, the bacterial probe was approached toward a fungal cell. Force maps of 16x16 pixels were recorded on the germ tubes using a maximum applied force of 250 pN, and constant approach and retraction speeds of 1000 nm/s. For each condition, the interaction forces between at least 5 pairs of bacterial-fungal cells from independent cultures were measured. Histograms were generated by considering the force and the distance of the last rupture event for every curve.

### Mammalian Cell Damage Assay

HeLa cells were seeded at 10^4^ cells/well in flat bottom tissue-culture-treated 96-well plates (Nunclon Delta Surface, Thermo Scientific). Cells were incubated for 24 h at 37°C and 5% CO_2_. Overnight cultures of *C. albicans* were washed three times with PBS and added to the HeLa cells to a final concentration of 1 × 10^5^ cells per well. Plates were incubated for 24 h at 37°C and 5% CO_2_. The CyQUANT™ LDH Cytotoxicity Assay Kit (Invitrogen) was used for read-out. Shortly, 50 µl of the supernatant of all wells was transferred to a new flat bottom 96-well plate and 50 µl of reaction mixture was added. Plates were incubated in the dark for 30 min. Next, 50 µl of stopping buffer was added and the absorbance was measured at 490 and 680 nm. Positive and negative controls were included. The percentage of cytotoxicity compared to the positive control was calculated by subtracting the 680 nm values from the 490 nm values.

### *In Vitro* Phagocytosis Assay

Tissue-culture-treated 12-well plates were seeded with 3 × 10^4^ cells/ml of *C. albicans* in YNB with 0.5% glucose pH 7 and statically incubated for 3 h at 37°C to allow initial attachment and hyphae formation. Wells were washed once with PBS and 1 × 10^6^ cells/ml of GFP-expressing *S. aureus* were added. Plates were incubated for 90 min under gentle agitation at 37°C to allow bacterial attachment to the hyphae. Afterwards, wells were washed once with PBS and 1 × 10^5^ cells/ml of J774 murine macrophages in DMEM supplemented with 10% FBS were added to the plates. Plates were immediately placed under the microscope to visualize phagocytosis. Images were taken every 2 min for 1 h. Macrophages, *C. albicans* and *S. aureus* were visualized by imaging bright-field illumination and the GFP signal at 20x magnification on three randomly selected positions per condition. Images were analyzed using the ImageJ software (Scheres and Krom, 2016).

### *In Vivo* Oral Co-Infection Murine Model

Eleven-week-old female C57BL/6J mice (Janvier) were used for all experiments. The model is based on Kong et al. (2015), with small modifications. To suppress the oral bacterial flora, mice received 0.5 mg/ml of ampicillin in their drinking water one week prior to the experiments. Mice were rendered susceptible to oral candidiasis by subcutaneous administration of cortisone acetate (225 mg/kg body weight; low dosage) in the dorsum of the neck on day 1 and day 3 (or with 250 mg/kg body weight on day 1, day 3, and day 5; high dosage). To ensure that the total volume of 200 µl cortisone was taken-up, mice were put asleep shortly with isoflurane to perform the injections and cortisone is vortexed well at all times (Solis and Filler, 2012). On the second day, mice were anesthetized by intraperitoneal injection of 50 mg/kg of ketamine and 0.65 mg/kg of medetomidine. Animals were kept on 37°C heating pads and ophthalmic ointment was applied to avoid drying of the eyes. Animals were orally inoculated by placing calcium alginate swabs sublingually for 1 h. These swabs were saturated for 10 min in a *C. albicans* suspension of 1 × 10^7^ cells/ml of in PBS. After infection, the anesthesia was reversed by an intraperitoneal injection of 0.5 mg/kg atipamezole. On day 4 the same was done with 6 × 10^6^ cells/ml of *S. aureus*. In addition, *S. aureus* was added to the drinking water in the same concentration. Control groups infected with *C. albicans* or *S. aureus* alone were included in the study. Animals were weighted and monitored daily and sacrificed by cervical dislocation on day 7. Tissues were harvested, homogenized and plated for enumeration of CFU on bacterial and yeast chromogenic media (CHROMagar) for *C. albicans* and *S. aureus*.

### Whole Blood Analysis

Mice blood samples were collected by cardiac puncture at the experimental endpoint and dipotassium ethylenediamine tetra-acetic acid (EDTA) was used as an anticoagulant. Immediately after collection, whole blood samples were analyzed using the ADVIA 2120i hematology analyzer (Siemens) to assess different hematological parameters.

### Histology

Tongues were fixed overnight in 4% paraformaldehyde, subsequently washed with PBS and further processed for histopathology. The sections were stained with Gram staining or PAS staining and examined with light microscopy.

### Statistical Analysis

Three independent biological replicates were used in all experiments. The graphs display the average values and the standard error of the mean (SEM). The statistical method used is clarified in the figure legend: *P < 0.05; **P < 0.01; ***P < 0.001 and ****P < 0.0001.

### Ethics Statement

All animal experiments were performed in accordance with the KU Leuven animal care guidelines and were approved by the Ethics Committee of the KU Leuven (project number P184/2017).

## Results

### *C. albicans* Als1 and Als3 Adhesins Mediate the Interaction With *S. aureus*

Previous studies showed that the *C. albicans* Als3 protein partially contributes to the interaction with *S. aureus* (Peters et al., 2012b). However, adherence of *S. aureus* to *C. albicans* hyphae still occurs to some extent when *ALS3* is deleted. We hypothesize that this is likely due to other cell surface molecules, such as Als1. Therefore, we have constructed *als1*Δ/Δ, *als3*Δ/Δ and *als1 als3* ΔΔ/ΔΔ mutant strains in the *C. albicans* SC5314 background. In the remainder of the manuscript we will refer to them as *als1*, *als3* or *als1 als3* mutants. No difference in growth was observed at 30°C and 37°C compared to the wild type strain (**Supplementary Figure 1**). As a control, adhesion and biofilm forming capacity of the different *C. albicans* strains was tested in the absence of bacteria in 96-well plates. *C. albicans* cells were statically incubated at 37°C for 90 min to allow adhesion, or for 24 h to allow biofilm formation. Only the *als1 als3* mutant strain was impaired in adhesion (**Supplementary Figure 2A**) and both the *C. albicans als3* and *als1 als3* strain were strongly impaired in biofilm formation compared to the wild type (**Supplementary Figure 2B**).

**Figure 1:**
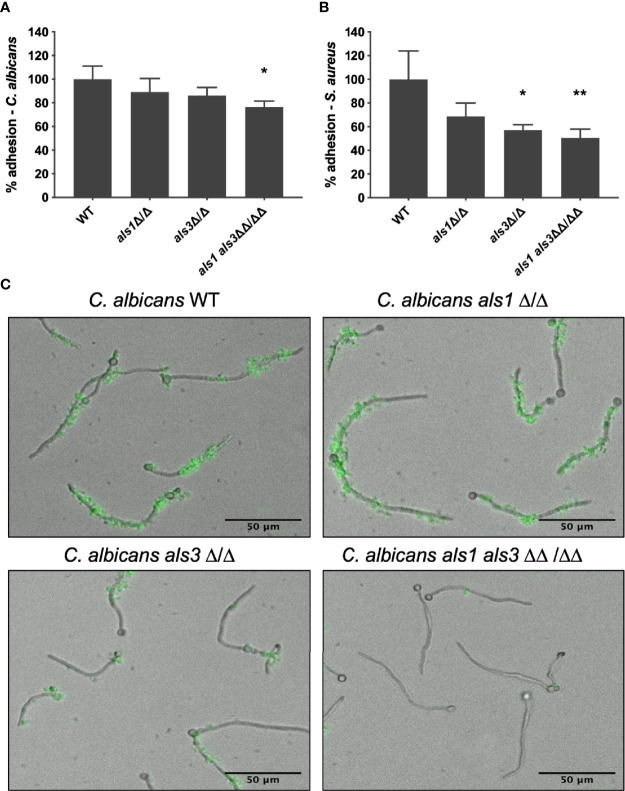
*C. albicans* Als3 and Als1 adhesins are responsible for the interaction with *S. aureus*. Relative percentage of adhesion of the *C. albicans* deletion strains **(A)** and *S. aureus* **(B)** in an *in vitro* mixed species static adhesion assay compared to the *C. albicans* wild type. Statistical analysis was conducted using one-way analysis of variance (ANOVA) with Bonferroni correction (*P < 0.05 and **P < 0.01). **(C)** Visual representations of GFP-labeled *S. aureus* adhesion to growing hyphae of the different *C. albicans* strains in an *in vitro* dynamic adhesion assay using a Bioflux system.

To verify whether Als1 plays an additional role in adhesion of *S. aureus* to *C. albicans*, a static *in vitro* mixed species adhesion assay was performed. Following 90 min of *C. albicans* adhesion, *S. aureus* cells were added to allow attachment of the bacterial cells. Results demonstrated no difference in adhesion of the *C. albicans* single deletion strains to the plastic surface, compared to the wild type strain. However, the adhesion ability of the *C. albicans als1 als3* double mutant strain was significantly reduced by almost 25% (**Figure 1A**). *S. aureus* adhered significantly less to the *C. albicans als3* and *als1 als3* deletion strains while there was no significant difference in adhesion to the *C. albicans als1* strain (**Figure 1B**). These results confirm that Als3 is important for the interaction with *S. aureus*. However, due to the inherently lower adhesion of the double deletion *C. albicans* strain to the plastic of the well plate, it is not feasible to conclude that deletion of *ALS1* has an additional effect on *S. aureus* adhesion. Therefore, an *in vitro* dynamic adhesion assay using a Bioflux system was performed. *C. albicans* cells were introduced in the channel and allowed to adhere to the surface and form germ tubes and hyphae. Subsequently, GFP-expressing *S. aureus* cells were flowed through the channel and adhesion was imaged over time. From these images (**Figure 1C**) and the calculated mean fluorescence intensity (**Supplementary Figure 3**), we observed no difference in the ability of *S. aureus* to adhere to the hyphae of the *C. albicans als1* strain compared to the wild type. Furthermore, we confirmed that the adhesion of *S. aureus* to the hyphae of the *C. albicans als3* strain was reduced, but not completely abolished. However, adhesion of *S. aureus* to the *C. albicans als1 als3* strain was even further reduced, indicating a plausible additional role for Als1 in supporting the physical interaction with *S. aureus*.

### Deletion of *ALS1* and *ALS3* Reduces the Adhesion Force Between *C. albicans* and *S. aureus*

**Figure 2:**
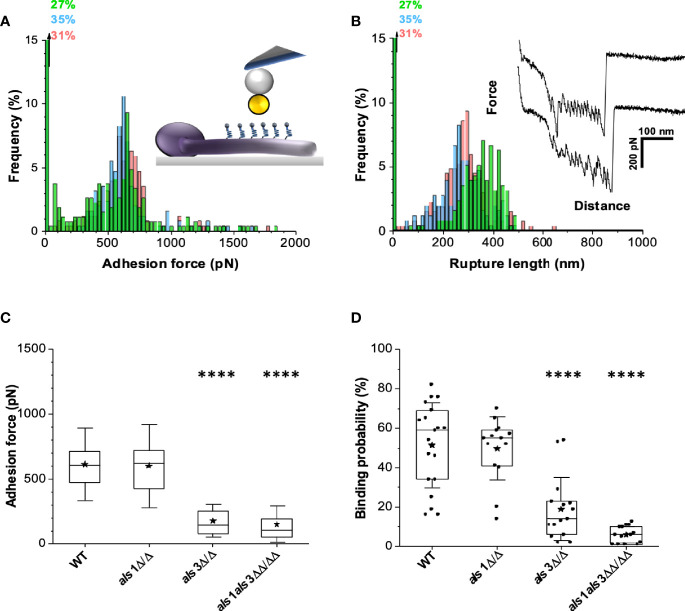
Deletion of *ALS3* and *ALS1* reduces the adhesion force between *C. albicans* and *S. aureus*. **(A)** Adhesion force histograms (Inset: schematic representation of the experiment.) and **(B)** rupture length histograms with representative retraction force profiles (inset) obtained by recording force-distance curves between *S. aureus* and *C. albicans* wild type. Adhesion force **(C)** and adhesion frequency **(D)** for single-cell force spectroscopy experiments obtained by probing the interaction between multiple pairs of *S. aureus*-*C. albicans* cells. Stars are the mean values, lines are the medians and boxes the 25-75% quartiles. Statistical analysis was conducted using one-way ANOVA with Bonferroni correction (****P < 0.0001).

To quantify bacterial-fungal adhesion forces, single-cell force spectroscopy (SCFS) was performed using a non-invasive protocol that ensures truly quantitative cell-cell interaction measurements (Beaussart et al., 2014; Xiao and Dufrene, 2016). A single bacterial cell was attached to a polydopamine-coated colloidal atomic force microscopy (AFM) probe and approached toward the germ tube of a fungal cell, immobilized on a hydrophobic substrate (**Figure 2A**, inset). This enabled us to quantify the adhesion forces between single bacterial and fungal cells. Force-distance curves recorded for *S. aureus* adhesion to *C. albicans* wild type germ tubes (**Figure 2B**, inset) featured adhesion events of 625 pN ± 286 pN (mean + standard deviation (s.d.), n = 541 adhesive force curves from 3 cell pairs) (**Figure 2A**) and with rupture lengths of 300 ± 91 nm (binding frequency ~70%) (**Figure 2B**). Similar data were obtained in 15 other cell pairs (**Figure 2C**). Adhesion forces did not substantially vary along the germ tubes, indicating their properties were homogeneous. Numerous force profiles featured sawtooth patterns with multiple force peaks of around 500 pN, which we attribute to the sequential unfolding of the tandem repeat domains of Als proteins in light of earlier force experiments. The 300 nm length is also consistent with the length of fully unfolded Als proteins (Beaussart et al., 2013).

To confirm that Als3 and Als1 adhesins indeed play a role in adhesion of *S. aureus*, we probed germ tubes from the *C. albicans als1* and *als3* single and double deletion strains. While the adhesion forces of the *als1* strain hardly changed compared to the wild type (adhesion events of 634 pN ± 273 pN and rupture lengths of 339 ± 94 nm; n = 462 adhesive force curves from 3 cell pairs) (**Supplementary Figures 4A, B****)**, the *als3* strain (**Supplementary Figure 4C**) and the *als1 als3* strain (**Supplementary Figure 4D**) showed a dramatic drop of adhesion force (198 pN ± 147 pN; n = 85 from 3 cell pairs; 198 pN ± 110 pN n = 59 from 3 cell pairs respectively). The average binding frequency was 10% for the *als3* strain and only 7% for the *als1 als3* double mutant strain (**Figure 2D**). These results further support that both adhesins are required for adhesion of *S. aureus*, though Als3 is crucial for stable anchoring as there is no statistical difference between the *als3* and *als1 als3* strain.

### *C. albicans* Als1 and Als3 are Required for *S. aureus* Dissemination

**Figure 3:**
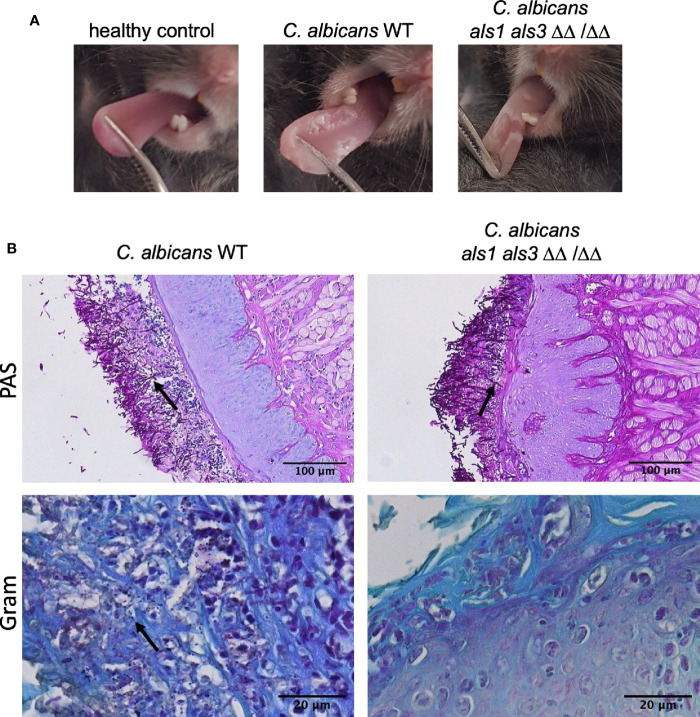
Mouse tongue co-infection images. **(A)** Clinical representation of *C. albicans* infection on the mouse tongues. A healthy control tongue and tongues of mice infected with *C. albicans* wild type and *C. albicans als1 als3* are shown. **(B)** Histopathology images of mice tongue tissue sections stained by PAS or Gram staining. Mice were co-infected with *C. albicans* wild type or *als1 als3* strain. Arrows indicate invasive *C. albicans* hyphae for the PAS-stained images and the presence of *S. aureus* on Gram-stained images.

**Figure 4:**
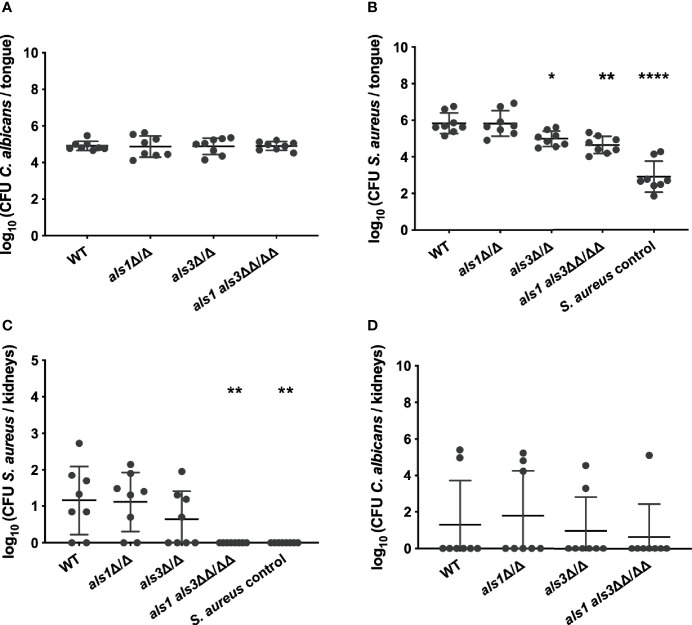
*C. albicans* Als3 and Als1 are crucial for bacterial dissemination. CFU of *C. albicans* **(A)** or *S. aureus* **(B)** retrieved from the tongues of co-infected mice. CFU of *C. albicans* **(C)** or *S. aureus* **(D)** retrieved from the kidneys of mice co-infected with the different *C. albicans* strains. A control is shown for mice only infected with *S. aureus*. Statistical analysis was conducted using one-way ANOVA with Bonferroni correction (*P < 0.05; **P < 0.01 and ****P < 0.0001).

While previous experiments were conducted *in vitro*, expression of *ALS1* might be more important *in vivo*. Since an *ALS3* deletion strain is still able to form biofilms *in vivo*, it was proposed that Als1 compensates for the loss of Als3 *in vivo* (Nobile et al., 2006). Therefore, we tested these deletion strains in the oral co-infection murine model. In summary, mice are rendered susceptible to oral candidiasis by administration of cortisone acetate (225 mg/kg body weight on day 1 and day 3). On the second day, animals are orally infected with *C. albicans* and two days later with *S. aureus*. Five days post *C. albicans* infection, organs were plated for enumeration of colony forming units (CFU) and tongue tissues were subjected to histopathology. Similar lesions on the tongues were observed for animals infected with the *C. albicans* wild type or the different deletions strains (**Figure 3A**). Microscopic analysis of tongue tissue sections stained by Periodic acid-Schiff (PAS) revealed *C. albicans* hyphal penetration of the superficial epithelial layer concomitant of an inflammatory response for both the wild type and *als1 als3* strain (**Figure 3B**). Gram-stained tissue sections showed the presence of *S. aureus* within the epithelium when co-infected with *C. albicans* wild type, however, the presence of *S. aureus* was less obvious for the *C. albicans als1 als3* strain (**Figure 3B**). There were no significant differences in fungal recovery between the strains (**Figure 4A**). Significantly less *S. aureus* was recovered from the tongues of the animals only infected with *S. aureus* compared to those co-infected with *C. albicans* (**Figure 4B**). There was no difference in bacterial load recovered from the tongues of mice co-infected with the *C. albicans als1* strain, however, a significant reduction was observed for the *C. albicans als3* and *als1 als3* strains compared to the wild type (**Figure 4B**). In 75% of the animals, *S. aureus* was able to disseminate to the kidneys when co-infected with the *C. albicans* wild type or *als1* strain (**Figure 4C**). Bacterial dissemination was reduced to 50% of the cases when co-infected with the *C. albicans als3* strain and no dissemination was observed when co-infected with the *C. albicans als1 als3* strain. There was also no bacterial dissemination in animals only infected with *S. aureus*. Although loss of both Als1 and Als3 has a profound effect on the dissemination of *S. aureus*, this is less clear for the presence of *S. aureus* on the tongue as there is no statistical difference between the *als3* and *als1 als3* strain. We observed dissemination of *C. albicans* to the kidneys in some animals of all groups. There was no significant difference between the *C. albicans* wild type or the different deletions strains (**Figure 4D**). These findings indicate that *C. albicans* Als1 and Als3 adhesins are crucial for *S. aureus* to disseminate.

### Bacterial Dissemination Depends on the Level of Immunosuppression

**Figure 5:**
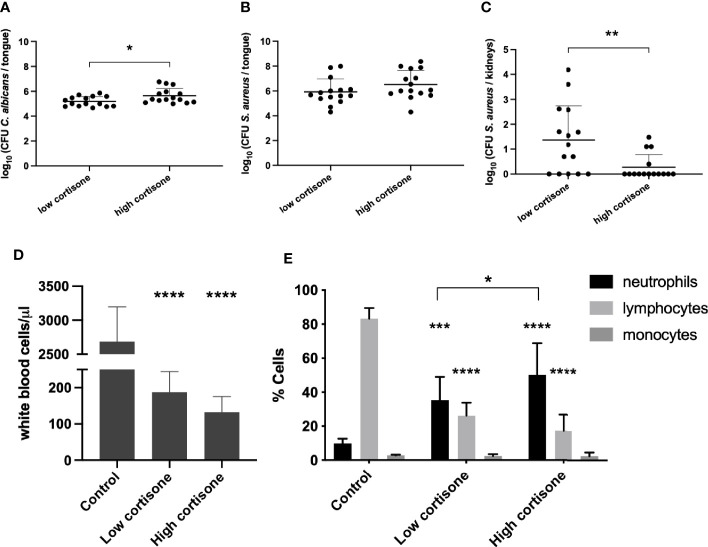
Effect of immunosuppression in an *in vivo* oral co-infection mouse model. CFU of *C. albicans* **(A)** and *S. aureus* **(B)** retrieved from the tongues of mice treated with a low or high cortisone dosage. **(C)** CFU of *S. aureus* retrieved from the kidneys of mice treated with a low or high cortisone dosage. Statistical analysis was conducted using an unpaired Student’s t-test (*P < 0.05 and **P < 0.01). **(D)** The total white blood cells measured in the whole blood. Statistical analysis was conducted using one-way ANOVA with Bonferroni correction. **(E)** Percentages of lymphocytes, monocytes and neutrophils from the total white blood cells measured in the whole blood from control mice or mice treated with a low or high cortisone dosage. Statistical analysis was conducted using two-way ANOVA with Bonferroni correction (*P < 0.05; ***P < 0.001 and ****P < 0.0001).

To investigate a possible role for the host immune response in bacterial dissemination after *C. albicans* colonization, we examined the effect of the immunosuppression in the oral co-infection model. Administration of cortisone is critical for *C. albicans* colonization of the oral cavity since mice which are not immunosuppressed will not develop OPC (Solis and Filler, 2012). To optimize the immunosuppression level for the *in vivo* co-infection experiments, we comparatively tested the following concentrations of cortisone: two injections of 225 mg/kg bodyweight (low dosage) and three injections of 250 mg/kg bodyweight (high dosage). The high dosage of immunosuppression resulted in more severe lesions on the tongues caused by *C. albicans*. Consistently, a higher fungal burden was recovered from the tongues of infected mice that were immunosuppressed with the higher dosage (**Figure 5A**). Slightly, yet not significantly, higher levels of *S. aureus* were recovered from mice treated with the higher cortisone dosage (**Figure 5B**). Interestingly, we observed significantly less dissemination of *S. aureus* to the kidneys when mice were administered the high dosage of immunosuppression (**Figure 5C**), indicating that high levels of immunosuppression inhibit bacterial dissemination. In addition, we observed that cortisone treated mice have significantly smaller spleens and cervical lymph nodes, possibly due to severe immune cell depletion (**Supplementary Figures 5A, B****)**. To confirm these speculations, whole blood samples from healthy control mice or mice treated with a low or high cortisone dosage were analyzed. Results demonstrated a significant decrease in total white blood cells in immunocompromised mice, supporting a severe cell depletion (**Figure 5D**). Additionally, a decrease in number of blood lymphocytes and increase in neutrophils proportional to cortisone dosage were observed (**Figure 5E**). These results strongly support that the host immune response plays a crucial role in the mechanism of *S. aureus* dissemination.

### *C. albicans* Als1 and Als3 Adhesins Ensure Bacterial Uptake by Phagocytic Immune Cells

**Figure 6:**
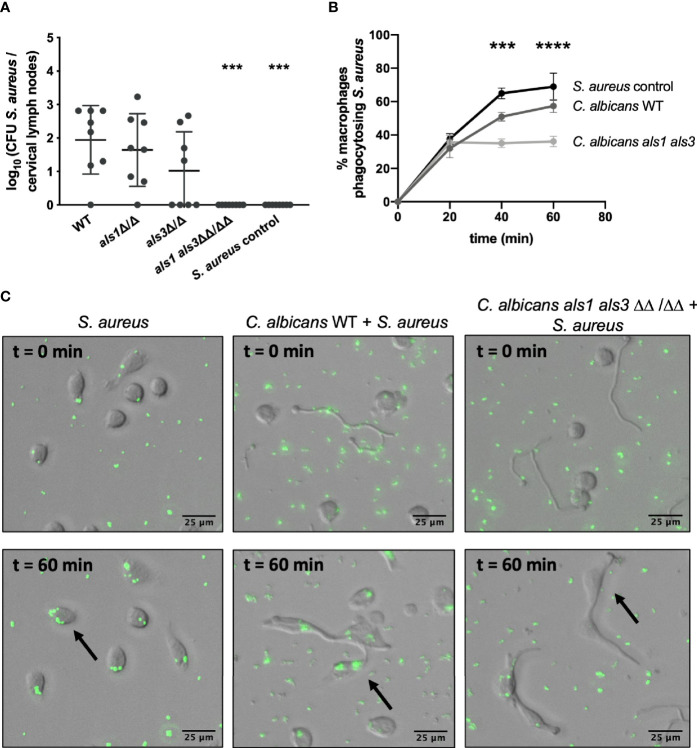
The interaction with *C. albicans* ensures bacterial uptake by phagocytic immune cells. **(A)** CFU of *S. aureus* retrieved from the cervical lymph nodes of mice co-infected with the different *C. albicans* strains. **(B)** Percentage of macrophages phagocytosing *S. aureus* after 0, 20, 40 and 60 min of phagocytosis. Statistical analysis was conducted using two-way ANOVA with Bonferroni correction (***P < 0.001 and ****P < 0.0001). **(C)** Visual representations are shown of the phagocytosis assays of GFP-labeled *S. aureus* alone or in combination with the *C. albicans* wild type and *als1 als3* strain at 0 and 60 min after addition of macrophages. Arrows indicate the uptake of bacteria inside macrophages.

Findings so far identified two requirements for *C. albicans*-mediated bacterial dissemination. Firstly, adhesion of *S. aureus* to *C. albicans* mediated by both Als1 and Als3 adhesins and secondly, the host immune response. As there is a possible role for host phagocytic immune cells in this process, using the murine co-infection model, we determined the CFU of *S. aureus* in the cervical lymph nodes which drain from the oral cavity (Allison et al., 2019). No viable *S. aureus* was retrieved from the lymph nodes when infected with the *als1 als3* mutant strain whereas this was the case for all other strains, indicating that Als1 and Als3 are required for *S. aureus* to be transported from the tongues to the lymph nodes, possible by phagocytic immune cells (**Figure 6A**). To elucidate why *C. albicans* Als1 and Als3 are required in this process, we performed *in vitro* phagocytosis assays with mixed *C. albicans* – *S. aureus* cultures. *C. albicans* cells were statically incubated to allow initial attachment and hyphae formation, then GFP-expressing *S. aureus* cells were added to allow bacterial attachment. Following incubation, murine macrophages were added and phagocytosis was visualized immediately. A control with no *C. albicans* cells included, demonstrated that macrophages cultured with *S. aureus*, efficiently engulf the surrounding bacteria (**Figures 6B, C****)**. When macrophages were co-cultured with *C. albicans*, they were highly attracted toward the *C. albicans* hyphae and importantly, they preferentially engulfed the bacteria attached to the *C. albicans* hyphae. Since bacterial attachment is strongly impaired for the *C. albicans als1 als3* mutant strain, we show that significantly fewer macrophages were phagocytosing *S. aureus* cells when co-cultured with the *C. albicans als1 als3* strain compared to the wild type strain (**Figures 6B, C****)**. As a control, we calculated the mean fluorescence intensity in each image and observed no significant difference in the amount of fluorescent *S. aureus* present (**Supplementary Figure 6A**). In addition, we confirmed that macrophage attraction was not impaired for the *C. albicans als1 als3* strain compared to the wild type (**Supplementary Figure 6B**). These results suggest that *S. aureus* adherence to *C. albicans* hyphae *via* Als1 and Als3 adhesins increases phagocytosis of *S. aureus* by macrophages and thereby provides an opportunity for dissemination. However, it is important to note that in the *in vivo* situation, fewer bacteria are present when co-infected with the *als1 als3* strain which could explain the reduction in *S. aureus* in the lymph nodes independently from phagocytosis by macrophages.

### Bacterial Dissemination Depends on the Secretion of Candidalysin

**Figure 7:**
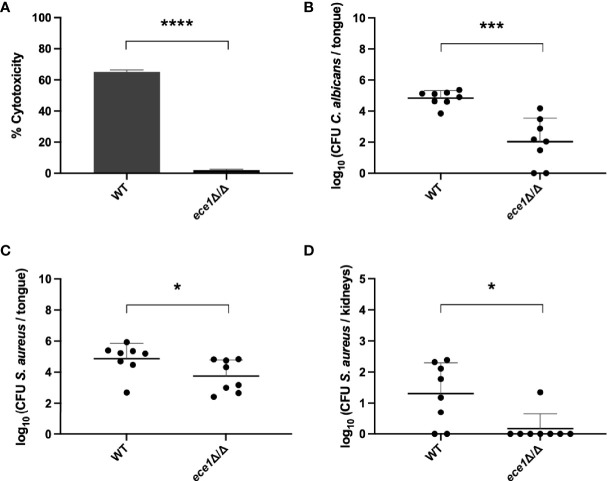
The role of *C. albicans ECE1* in an *in vivo* oral co-infection mouse model. **(A)** Percentage of cytotoxicity to mammalian cells caused by the *C. albicans* wild type or *ece1* strain. CFU of *C. albicans* **(B)** and *S. aureus* **(C)** retrieved from the tongues of mice infected with the *C. albicans* wild type or *ece1* strain. **(D)** CFU of *S. aureus* retrieved from the kidneys of mice infected with the *C. albicans* wild type or *ece1* strain. Statistical analysis was conducted using an unpaired Student’s t-test (*P < 0.05; ***P < 0.001 and ****P < 0.0001).

To confirm that activation of the host immune response plays a role in the mechanism of *S. aureus* dissemination, we studied the involvement of candidalysin. Candidalysin is the best-known *C. albicans* peptide responsible for activation of the immune response and immune cell recruitment (Naglik et al., 2019). It is one of the eight peptides which are produced after processing of the Ece1 protein. Therefore, we generated the *C. albicans ECE1* deletion strain (further referred to as *ece1* strain) in the SC5314 background. Since it has been reported that candidalysin is also involved in cell damage (Moyes et al., 2016), we tested the *ece1* strain for cell damage to mammalian cells *in vitro*. As expected, we observed significantly less damage caused by the *ece1* strain compared to the wild type (**Figure 7A**). Consistent with these findings, when the *ece1* strain was tested in the co-infection mouse model, only small oral lesions were seen 5 days after infection with the *C. albicans ece1* strain, indicating that the infection is less severe compared to animals infected with the wild type strain. A significantly smaller fungal load (**Figure 7B**) and significantly less *S. aureus* (**Figure 7C**) were recovered from the tongues of animals infected with the *C. albicans ece1* deletion strain. Furthermore, bacterial dissemination was observed in only one animal when co-infected with the *C. albicans ece1* strain (**Figure 7D**). Interestingly, *in vitro* adhesion assays between *S. aureus* and the *C. albicans ece1* strain showed no significant difference in the capacity of *S. aureus* to adhere to the *ece1* deletion strain compared to the wild type strain (**Supplementary Figures 7A, B**). Combined, these findings demonstrate that Ece1 is required for *C. albicans* colonization, immune activation and subsequent bacterial dissemination.

## Discussion

Polymicrobial interactions within biofilm-associated infections can enhance the virulence of the pathogens and thereby greatly influence the outcome of these infections (Harriott and Noverr, 2011; Carolus et al., 2019). Although *C. albicans* and *S. aureus* are normal commensals of most healthy humans, they are also opportunistic pathogens. We previously showed that oral infection with *C. albicans* predisposes for a secondary systemic infection of *S. aureus* in mice (Kong et al., 2015). In this study, we unraveled the host and pathogen mechanisms mediating bacterial dissemination. In conclusion, we show that there are two prerequisites for bacterial dissemination to occur: attachment of *S. aureus* to *C. albicans* Als1 and Als3 adhesins and the host immune response.

The *C. albicans* Als3 adhesin was known to partially mediate the interaction with *S. aureus* and was shown to be important in the oral co-infection model yet the additional molecules which contribute to this interaction were not yet identified (Peters et al., 2012b; Schlecht et al., 2015). We have previously shown that Als3 as well as Als1 contribute to the interaction of *C. albicans* with *Staphylococcus epidermidis* and the involvement in the interaction with *Streptococcus gordonii* was shown by others (Beaussart et al., 2013; Hoyer et al., 2014). By deleting these two proteins in this study, we show that the physical interaction with *S. aureus* is further reduced, demonstrating that in addition to Als3, Als1 contributes to the co-adherence of these species. However, there was no significant reduction in *S. aureus* presence on the tongue when infected with the *als1 als3* strain, compared to the *als3* single deletion strain. Importantly, deletion of both adhesins completely prevented bacterial dissemination in the oral co-infection mouse model, while this was not the case for the als3 strain, supporting the idea that the presence of both adhesins is crucial for *S. aureus* dissemination.

Remarkably, the level of immunosuppression appeared to play an important role in the mechanism of bacterial dissemination. We found that a certain level of immunosuppression is necessary for the development of oral candidiasis, however, strongly immunosuppressed mice showed less dissemination of *S. aureus*. In mice treated with cortisone, severe cell depletion and an increased release of neutrophils in the blood were observed. This is called neutrophilia, which is also known to be accompanied with a decrease in migration of neutrophils to tissues (Ronchetti et al., 2018). In addition, cortisone acetate reduces the production of monocytes, such as macrophages, in the spleen and thereby indirectly prevents accumulation of these immune cells at infected tissue sites. Interestingly, when a range of cortisone dosages was tested, it was shown that lower dosages only delayed the development of immunity by inhibiting cell proliferation in the spleen. However, the higher dosages, completely inhibited infection-induced spleen cell-proliferation and thereby severely suppressing immunity (North, 1971). In this regard, we hypothesize that the low cortisone dosage only delays cell-mediated immunity and allows development of OPC, however, phagocytic immune cells will still engulf *S. aureus*, which gives an opportunity for *S. aureus* to disseminate. *S. aureus* was shown to be able to survive phagocytosis by macrophages and neutrophils (Singh, 2017; Moldovan and Fraunholz, 2019). In addition, we show that the physical interaction with *C. albicans* Als1 and Als3 enhances bacterial uptake by host phagocytic immune cells, thereby linking the requirement of the physical interaction with the importance of the immune response. It is important to note that this is a simplification of the *in vivo* situation in which a wide variety of immune cells and other host factors are present. Further research should focus on the investigation of this process in the *in vivo* mouse model using non-invasive imaging techniques.

Recently, it was shown that during mucosal *C. albicans* infections, candidalysin, a cytolytic peptide secreted by the hyphal form during invasion, triggers a protective immune response by the host. After recognition of the peptide, epithelial signaling is triggered which initiates neutrophil recruitment and induces maturation of primary macrophages. In addition, candidalysin mediates host cell damage and is thereby important in mucosal infections (Moyes et al., 2016; Naglik et al., 2019; Rogiers et al., 2019). Our data show that Ece1, the protein from which candidalysin is produced, is required for *S. aureus* dissemination. However, Ece1 is processed to eight different peptides indicating that the effect is not certainly due to absence of the candidalysin peptide. In addition, it remains questionable whether the absence of bacterial dissemination is due to the lack of immune activation or the reduced colonization. Importantly, there is a reduction in *S. aureus* dissemination for the *C. albicans als1 als3* deletion strain, however this strain still secretes candidalysin. Similarly, there is a reduction in *S. aureus* dissemination for the *ece1* deletion strain, although there is no adhesion defect of *S. aureus*. This might indicate that these are two independent steps in the process of bacterial dissemination which are both required.

In conclusion, based on our findings, *C. albicans* Als3 and Als1 could be considered as potential interesting targets in the development of treatment strategies for polymicrobial infections. Interesting *S. aureus* associated-factors mediating the interaction with *C. albicans* were investigated previously (Schlecht et al., 2015). *S. aureus* fibronectin binding protein B (FnpB), a putative surface anchored protein (SasF) and a putative N-acetylmuramoyl-L-alanine amidase (Atl) were found to play a role in binding to *C. albicans* hyphae. Further research should focus on how these factors possibly bind *C. albicans* Als3 and Als1 and whether they are important for *S. aureus* dissemination. In addition, a possible role for candidalysin in mediating interspecies interactions will be of interest in further research. These findings highlight the complexity of interactions between pathogens and the host in polymicrobial infections, and identify a potential paradoxical role for immunosuppression in a clinical setting.

## Data Availability Statement

The original contributions presented in the study are included in the article/**Supplementary Material**. Further inquiries can be directed to the corresponding author.

## Ethics Statement

The animal study was reviewed and approved by Ethics Committee on animal experimentation of the KU Leuven (project number P184/2017).

## Author Contributions

Conceptualization: KV, MJ-R, GV, YD, BK, and PV. Methodology and investigation: KV, FV, MM-G, and LD. Formal analysis: KV, FV, MM-G, and EH. Writing (original draft): KV. Writing (review and editing): all authors. Funding acquisition: MJ-R, YD, and PV. Resources, YD, BK, and PV. Supervision: MJ-R, GV, YD, BK, and PV. All authors contributed to the article and approved the submitted version.

## Funding

KV was supported by a personal research grant (1181818N) from the Fund for Scientific Research Flanders (FWO). This work was supported by the FWO research community on biofilms (W000921N) and by the National Institute of Allergy and Infectious Diseases of the NIH under award number R01AI130170 (NIAID) to MJ-R. Work at the Université Catholique de Louvain was supported by the National Fund for Scientific Research (FNRS) and the Research Department of the Communauté Française de Belgique (Concerted Research Action). YD is a Research Director at the FNRS.

## Conflict of Interest

The authors declare that the research was conducted in the absence of any commercial or financial relationships that could be construed as a potential conflict of interest.
