# Supplementary material for: Adhesion of Staphylococcus aureus to Candida albicans During Co-Infection Promotes Bacterial Dissemination Through the Host Immune Response
Source: Front Cell Infect Microbiol. 2021 Feb 2;10:624839. doi: 10.3389/fcimb.2020.624839 (PMC7884861; doi:10.3389/fcimb.2020.624839)
Supplement: Supplementary file 1 [file DataSheet_1.pdf]

Supplementary Material

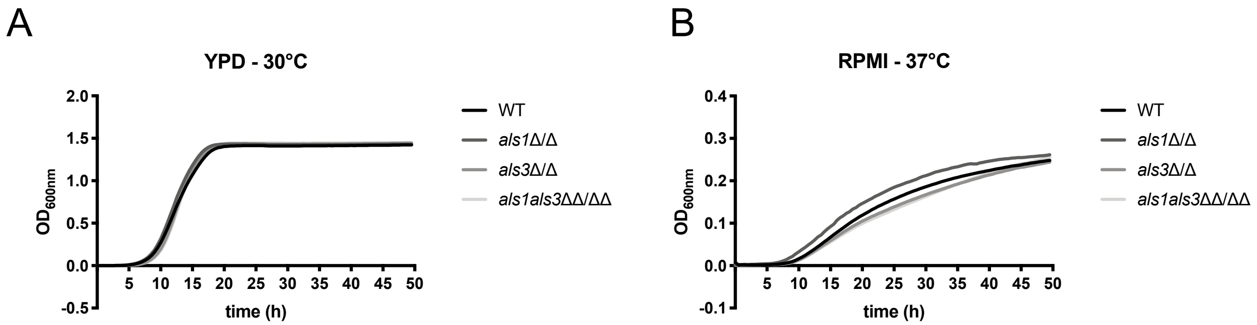

Supplementary Figure 1. Growth curves of *C. albicans* deletion strains. Growth curves of the different *C. albicans* deletion strains and the wild type strain in (A) YPD at 30°C and (B) RPMI medium at 37°C.

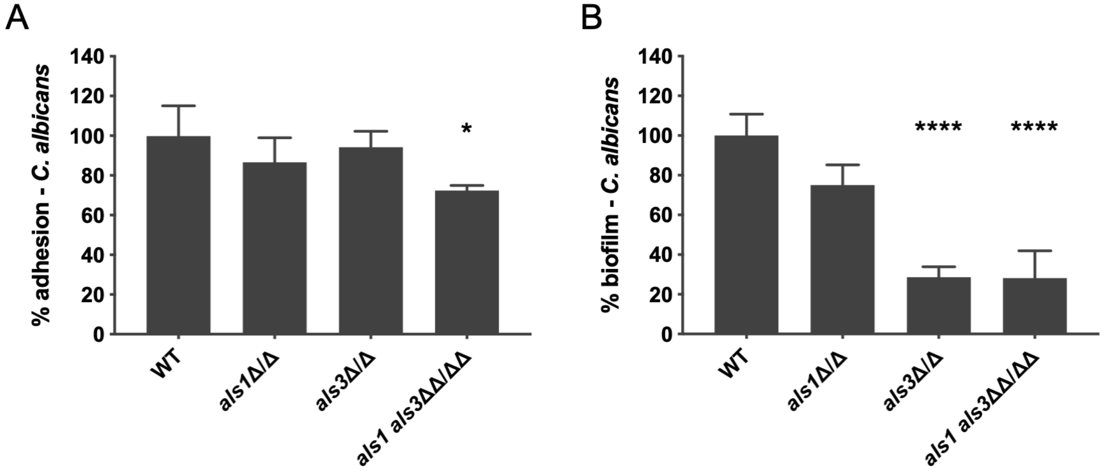

Supplementary Figure 2. *In vitro* adhesion and biofilm formation of *C. albicans* deletion strains. Relative percentage of adhesion (A) and biofilm formation (B) of *C. albicans* deletion strains compared to the wild type strain in an *in vitro* static assay. Statistical analysis was conducted using one-way ANOVA with Bonferroni correction (*, $P<0.05$ and ****, $P<0.0001$).

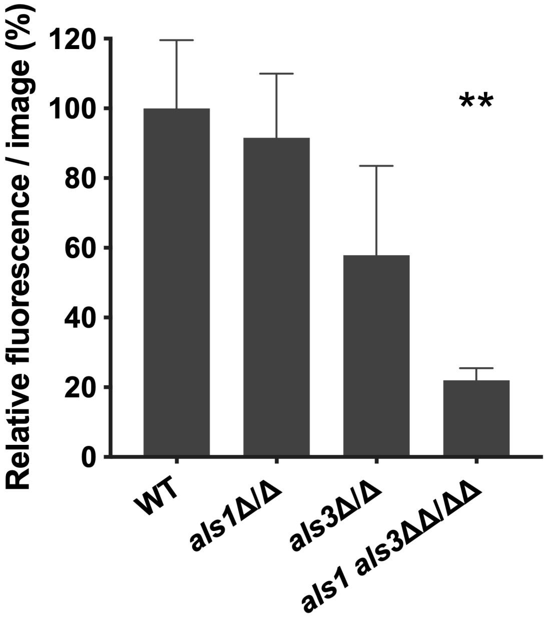

Supplementary Figure 3. Fluorescence intensity of *S. aureus* adhesion to *C. albicans.* The relative mean fluorescence intensity per image of GFP-labelled *S. aureus* adhesion to growing hyphae of the different *C. albicans* strains compared to the wild type in an *in vitro* dynamic adhesion assay using a Bioflux system. Statistical analysis was conducted using one-way ANOVA with Bonferroni correction (**, P<0.01).

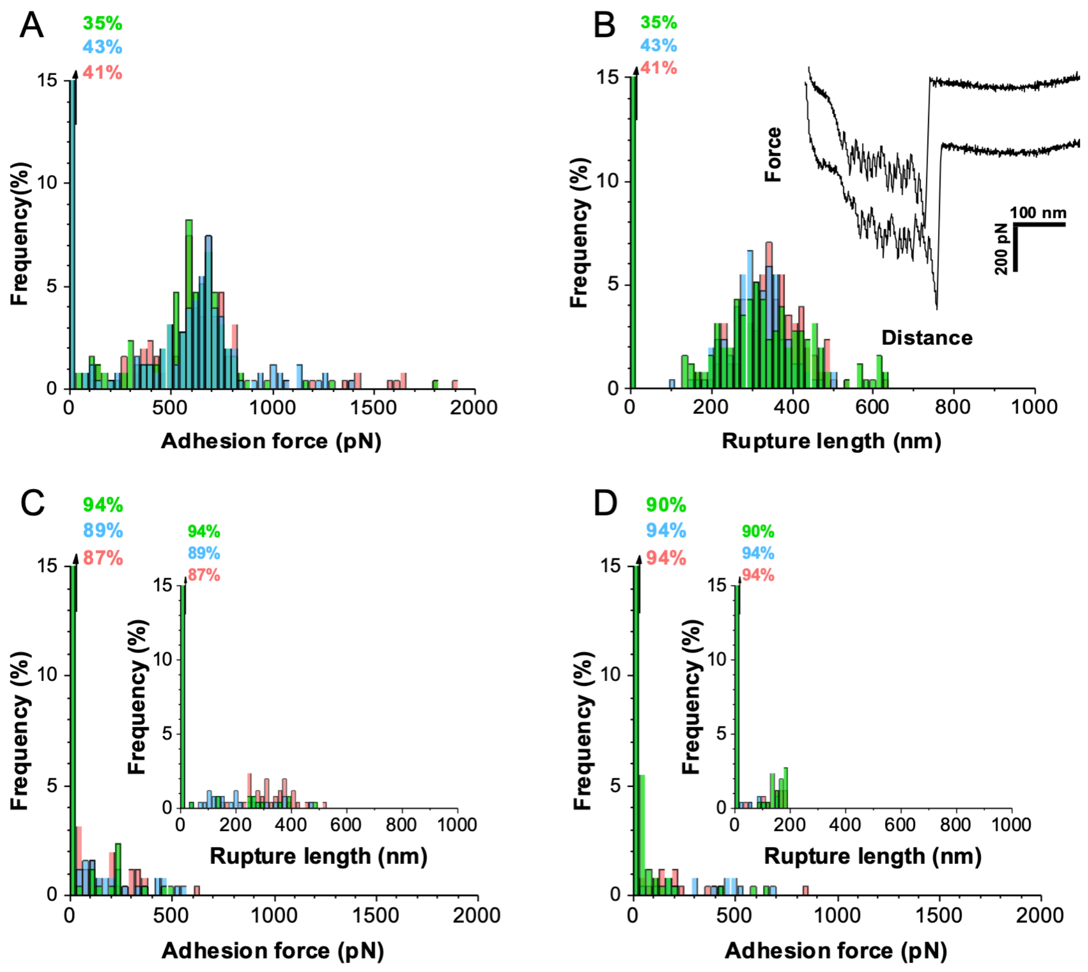

Supplementary Figure 4. Single-cell force spectroscopy of *S. aureus*-*C. albicans* interactions. Force data (A) and rupture length data (B), with representative retraction force profile (inset), obtained for the interaction between *S. aureus* and *C. albicans als1* mutant strain. Adhesion force (C) and rupture length distribution (inset) obtained for the interaction between *S. aureus* and *C. albicans als3* mutant strain. Adhesion force (D) and rupture length distribution (inset) obtained for the interaction between *S. aureus* and *C. albicans als1 als3* strain.

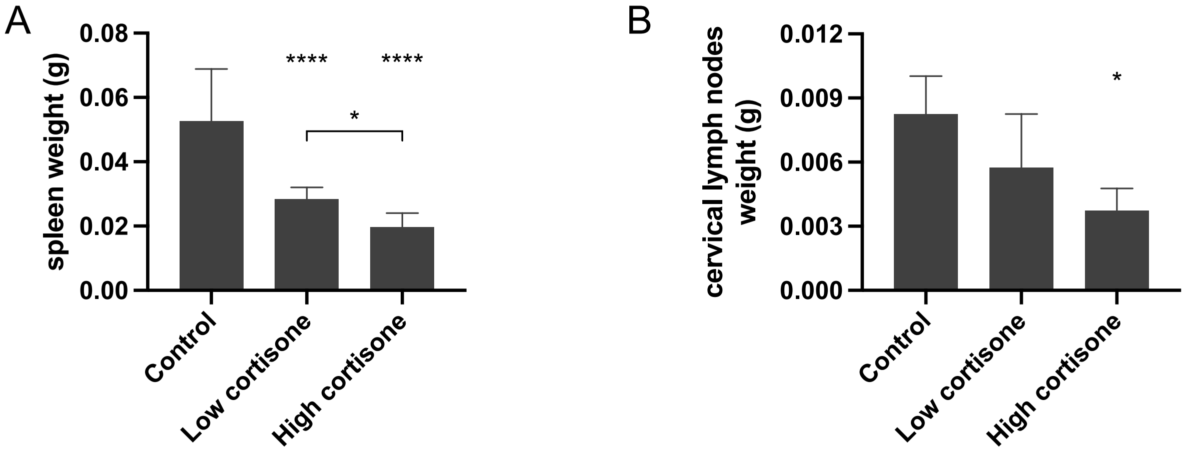

Supplementary Figure 5. Weight of spleens and cervical lymph nodes. The relative weight of spleens (A) and cervical lymph nodes (B) from mice treated with a low or high cortisone dosage compared to a control. Statistical analysis was conducted using one-way ANOVA with Bonferroni correction (*, $P<0.05$ and ****, $P<0.0001$).

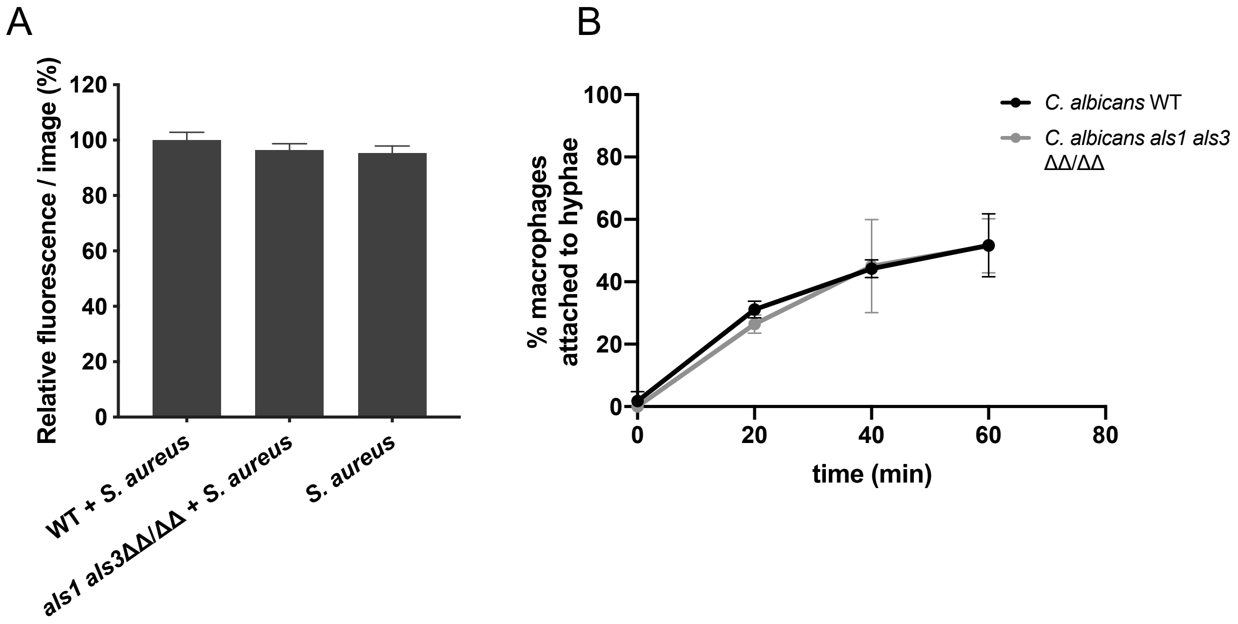

Supplementary Figure 6. Percentage of fluorescent *S. aureus* and in vitro phagocytosis of *C. albicans.* (A) The relative mean fluorescence intensity per image of GFP-labelled *S. aureus* of the *C. albicans als1 als3* strain compared to the wild type in the *in vitro* phagocytosis experiments. Statistical analysis was conducted using one-way ANOVA with Bonferroni correction. (B) Percentage of macrophages attached to the *C. albicans* hyphae after 0, 20, 40 and 60 min of phagocytosis. Statistical analysis was conducted using two-way ANOVA with Bonferroni correction.

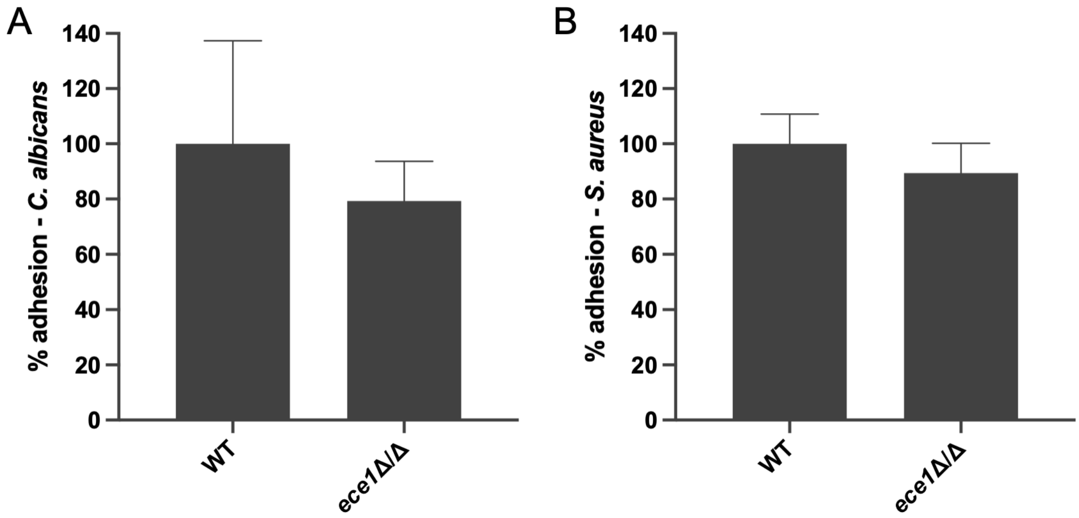

Supplementary Figure 7. *In vitro* static adhesion assay of *S. aureus* to *C. albicans ece1*Δ/Δ. Relative percentage of adhesion of the *C. albicans ECE1* deletion strain (A) and *S. aureus* (B) in an *in vitro* mixed species static adhesion assay compared to the *C. albicans* wild type. Statistical analysis was conducted using an unpaired t-test.

Supplementary Table 1. Primers used in this study.

**Primer name**	**Sequence 5' → 3'**
**Primers used for generating the *C. albicans* deletion strains**	
*ALS1*_upstream_Fw	AAGGGAACAAAAGCTGGGTACCGGGCCTCTTTCCTATCCGATAACC
*ALS1*_upstream_Rv	TATTCTCTAGAAAGTATAGGAACTTCCAATTGAGAGGAGGAAAGAG
*ALS1*_downstream_Fw	GAACTTCAGATCCACTAGTTCTAGAGCGTTATGATCACCTTTTTGGC
*ALS1*_downstream_Rv	CGACTCACTATAGGGCGAATTGGAGCTGCAATTCCGTGTGTCTAAAAG
*ALS3*_upstream_Fw	AAGGGAACAAAAGCTGGGTACCGGGCCTAACCCGCCTCAAATCAAG
*ALS3*_upstream_Rv	TATTCTCTAGAAAGTATAGGAACTTCCCTATCAGACCTCAATTCAAGG
*ALS3*_downstream_Fw	GAACTTCAGATCCACTAGTTCTAGAGCATACCATCATAGTCGCCTTTTTAG
*ALS3*_downstream_Rv	CGACTCACTATAGGGCGAATTGGAGCTGTCTTACTTTGGTGCATC
*ECE1*_upstream_Fw	AAGGGAACAAAAGCTGGGTACCGGGCCCTTCGTTATCAGTGGCTTCAT
*ECE1*_upstream_Rv	TATTCTCTAGAAAGTATAGGAACTTCCTCGAGTACTTCAATTCTGAGCCCTT
*ECE1*_downstream_Fw	GAACTTCAGATCCACTAGTTCTAGAGCGGCCGCTGGTGGTGTTGAATTGCTTT
*ECE1*_downstream_Rv	CGACTCACTATAGGGCGAATTGGAGCTCGGTTGCCATAAACAGTAGAA
